# Induced abortions of women living with HIV in Finland 1987–2019: a national register study

**DOI:** 10.1186/s12884-023-05430-x

**Published:** 2023-02-18

**Authors:** Mikaela Mutru, Pia Kivelä, Jukka Ollgren, Kirsi Liitsola, Mika Gissler, Inka Aho

**Affiliations:** 1 University of Helsinki, Biomedicum, Haartmaninkatu 8, 00014 Helsinki, Finland; 2 Department of Infectious Diseases, Helsinki University Hospital, Helsinki, Finland; 3 Finnish Institute for Health and Welfare, Helsinki, Finland; 4 Academic Primary Health Care Centre, Region Stockholm, Stockholm, Sweden; 5 Department of Molecular Medicine and Surgery, Karolinska Institutet, Stockholm, Sweden

**Keywords:** HIV infections, Induced abortion, HIV testing, Registries, Retrospective studies

## Abstract

**Background:**

Recent data on the rate and risk factors of induced abortion among women living with HIV (WLWH) are limited. Our aim was to use Finnish national health register data to 1) determine the nationwide rate of induced abortions of WLWH in Finland during 1987–2019, 2) compare the rates of induced abortions before and after HIV diagnosis over different time periods, 3) determine the factors associated with terminating a pregnancy after HIV diagnosis, and 4) estimate the prevalence of undiagnosed HIV at induced abortions to see whether routine testing should be implemented.

**Methods:**

A retrospective nationwide register study of all WLWH in Finland 1987–2019 (*n* = 1017). Data from several registers were combined to identify all induced abortions and deliveries of WLWH before and after HIV diagnosis. Factors associated with terminating a pregnancy were assessed with predictive multivariable logistic regression models. The prevalence of undiagnosed HIV at induced abortion was estimated by comparing the induced abortions among WLWH before HIV diagnosis to the number of induced abortions in Finland.

**Results:**

Rate of induced abortions among WLWH decreased from 42.8 to 14.7 abortions/1000 follow-up years from 1987–1997 to 2009–2019, more prominently in abortions after HIV diagnosis. After 1997 being diagnosed with HIV was not associated with an increased risk of terminating a pregnancy. Factors associated with induced abortion in pregnancies that began after HIV diagnosis 1998–2019 were being foreign-born (OR 3.09, 95% CI 1.55–6.19), younger age (OR 0.95 per year, 95% CI 0.90–1.00), previous induced abortions (OR 3.36, 95% CI 1.80–6.28), and previous deliveries (OR 2.13, 95% CI 1.08–4.21). Estimated prevalence of undiagnosed HIV at induced abortion was 0.008–0.029%.

**Conclusions:**

Rate of induced abortions among WLWH has decreased. Family planning should be discussed at every follow-up appointment. Routine testing of HIV at all induced abortions is not cost-effective in Finland due to low prevalence.

## Background

Before effective methods to prevent vertical transmission were discovered studies reported an increased proportion of pregnancies ending in induced abortion after HIV diagnosis [1, 2]. Development of antiretroviral treatment (ART) has reduced the risk of vertical transmission in European cohort studies from 15 to 20% in the pre-ART era to 10% with mono-ART and to less than 1% in the current era of combined ART (cART) [3–7]. Although over 80% of the nearly 50,000 women newly diagnosed with HIV in Europe in 2018 were fertile-aged [8], little is known about the rates and risk factors for induced abortions among women living with HIV (WLWH). Most data come from limited questionnaire studies with varying response rates [9–11]. In questionnaire studies on the general population, induced abortions have been significantly underreported [12–14]. The risk factors for WLWH having an induced abortion have included younger age, earlier year of HIV diagnosis, and transmission by intravenous drug use (IDU) [15].

In Finland, an indication is needed for induced abortion but 95% of abortions are performed on social indications [16]. Induced abortion is widely available and inexpensive in public health care. Since HIV testing at an induced abortion is currently not routinely recommended, the prevalence of undiagnosed HIV at the time of induced abortion is unknown.

The aim of the study was to use Finnish national health register data to 1) determine the nationwide rate of induced abortions of WLWH in Finland in 1987–2019, 2) compare the rates of induced abortions before and after HIV diagnosis over different time periods, 3) determine the factors associated with terminating a pregnancy after HIV diagnosis, and 4) estimate the prevalence of undiagnosed HIV at induced abortions to see whether routine testing should be implemented.

## Methods

This is a nationwide retrospective study combining data from several national registers. The study population consisted of all women who were diagnosed with or treated for HIV in Finland before 1 January 2020, were fertile-aged (15**–**49 years) between 1987 and 2019, and had a valid personal identification code (PIC; granted to all citizens and immigrants with a permit to stay at least 1 year). The women were identified from the Finnish HIV Quality of Care Register (FINHIV), which is described in detail elsewhere and consists of all people diagnosed with or treated for HIV in Finland since 1 January 1984 [17]. Sex in the register is reported predominantly based on the PIC which is sex-specific (categories female/male); generally this means sex assigned at birth.

Using the PIC as an identification, data from the FINHIV were combined with two national registers maintained in the Finnish Institute for Health and Welfare (THL): Register on Induced Abortions and Sterilizations, and Medical Birth Register. Reporting induced abortions, sterilizations, and births to their respective registers is mandatory by law, and the electronical information is available since 1983 (induced abortions), 1987 (sterilizations), and 1987 (births). The following variables were collected: from the FINHIV date of birth, date of death, date of HIV diagnosis, transmission route, country of birth, date of immigration, date of emigration, first CD4+ cell count and its date, date of AIDS diagnosis, hepatitis C antibodies (positive/negative), hepatitis B S-antigen (positive/negative), and date of latest contact to HIV care (e.g. date of latest HIV viral load); from the Register on Induced Abortions and Sterilizations date of abortion, gestational age at abortion, indication for abortion, and date of sterilization; from the Medical Birth Register date of delivery and gestational age at birth. Because only data on induced abortions in Finland were available and the immigration and emigration data in the FINHIV are incomplete, the estimated follow-up time was corrected for those not born in Finland with dates of first and latest recorded contact to health care. These were collected from the Care Register for Health Care and Register for Primary Health Care Visits (maintained at THL; earliest data from 1994 and 2011, respectively).

**Fig. 1:**
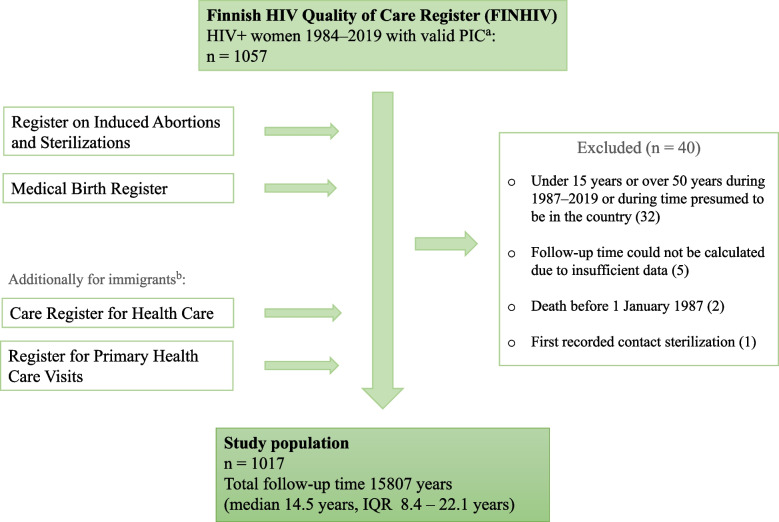
Formation of the study population. ^a^Personal Identification Code, given to all citizens or immigrants with permit to stay at least 1 year. ^b^Dates of first and last recorded contact to health care were collected from these registers and used as cut-off points for follow-up time for foreign-born women because immigration and emigration data in the FINHIV are incomplete

For women born in Finland, the start of the follow-up was defined as 1 January 1987 or the day they turned 15 years of age, whichever later. The end of the follow-up was defined as 31 December 2019, the date of turning 50 years of age or, when applicable, date of death, sterilization or emigration. For women not born in Finland, we used additional cut-off points to correct for the time before immigration and the increased rate of emigration: for the start of follow-up, they consisted of the date of immigration as recorded in the FINHIV or the first recorded health care contact (including the date of first delivery or induced abortion). Similarly, for the end of the follow-up the additional cut-off point was the last recorded date of health care contact. Formation of the study population is depicted in Fig. 1.

The date of HIV diagnosis is most often recorded as the date the first positive HIV antigen/antibody test was taken, and there is a delay of approximately 7 days before a positive result is confirmed. Arranging an induced abortion takes a minimum of 3 days. Therefore, an induced abortion was considered to have taken place before the HIV diagnosis (i.e., positive HIV test result could not have affected the decision to have an induced abortion) if the date of the abortion was before the date of HIV diagnosis, or the HIV diagnosis date was no more than 10 days prior to the date of abortion. If the HIV diagnosis date was earlier than 10 days prior to the date of abortion, the abortion was considered to have taken place after the HIV diagnosis.

A predictive multivariable logistic regression model was used to assess the factors associated with terminating a pregnancy, with all pregnancies ending in delivery or induced abortion in 1987–2019 included and clustering by person. An additional analysis focusing on the factors associated with WLWH having an induced abortion was done by constructing another model with time limited to 1998–2019 and to time and pregnancies that began after HIV diagnosis. The models were created by including all available variables and checking for interactions, and the final models were chosen by including variables and interactions terms based on significance in the model construction (*p* ≤ 0.05) and by comparison of Akaike and Bayesian Information Criteria between models.

To estimate the maximum prevalence of undiagnosed HIV at the time of induced abortion, we examined all induced abortions taking place 0–5 years before HIV diagnosis during 1987–2014. Additionally, we used the CD4-positive lymphocyte count at HIV diagnosis (if measured 0–90 days after HIV diagnosis) to assess whether the women had been infected at the time of the last induced abortion. The thresholds for approximating the time passed since transmission were based on calculations by Lodi et al. [18] where the median times for 25–30-year-old heterosexual women from seroconversion to CD4+ cell count dropping below 500 cells/μL, 350 cells/μL, and 200 cells/μL were 1.63, 5.66, and 10.71 years, respectively. We set the thresholds for probable minimum time as infected at 6 months for those with CD4+ cell count over 500 cells/μL, 1 year for those with CD4+ cell count 350–499 cells/μL, 3 years for those with 200–349 cells/μL, and 5 years for those under 200 cells/μL. The calculated maximum and minimum numbers of induced abortions with undiagnosed HIV were then compared to the total number of induced abortions in the time period as recorded in the Register of Induced Abortions and Sterilizations [19]. We also evaluated the prevalence for some subgroups: women not born in Finland, women with previous induced abortions, and women with previous deliveries. The total of induced abortions performed for these subgroups was estimated from THL open data [19] for previous pregnancies and from articles by Malin et al. [20] and Heino et al. [16] for women not born in Finland.

For statistical analysis, SPSS 27.0 (IBM, Chicago, Illinois, USA) and Stata 17 (StataCorp LLC, College Station, Texas, USA) were used.

## Results

The study consists of 1017 women described in Table 1. The total follow-up time was 15,807 years (median 14.5 years, IQR 8.4–22.1), with 6509 years before and 9297 years after HIV diagnosis. A total of 249 women (24.5% of the study population) had 396 induced abortions. The number of abortions per woman ranged from 1 to 6, with 154 (61.8%) having one abortion, 61 (24.5%) having two, and 34 (13.0%) having three or more. The indication for induced abortion was recorded in 386 cases (97.5%); social circumstances for 349 (90.4% of those recorded), under 17 years of age in 13 (3.4%), endangerment of the woman’s health in 11 (2.8%), fetal indication in 6 (1.6%), and other indication in 7 (1.8%).

**Table 1:** Characteristics of the study population

Population	All		Divided by year of HIV diagnosis^a^					
			1987–1997		1998–2008		2009–2019	
Number of women	**1017**		138	13.6%	413	40.6%	466	45.8%
Median age at HIV diagnosis	**30.7**		29.0		30.4		32.0	
Country of birth								
Finland	**428**	**42.1%**	97	70.3%	205	49.6%	126	27.0%
Other	**586**	**57.6%**	41	29.7%	208	50.4%	337	72.3%
Unknown	**3**	**0.3%**	0		0		3	0.6%
Diagnosed before immigration	**159**	**15.6%**	10	7.2%	45	10.9%	104	22.3%
Mode of transmission								
Sex	**809**	**79.5%**	118	85.5%	289	70.0%	402	86.3%
IDU^b^	**99**	**9.7%**	8	5.8%	81	19.6%	10	2.1%
Vertical^c^	**13**	**1.3%**	3	2.2%	8	1.9%	2	0.4%
Blood transfusion^c^	**11**	**1.1%**	2	1.4%	2	0.5%	7	1.5%
Unknown	**85**	**8.4%**	7	5.1%	33	8.0%	45	9.7%
Hepatitis C antibodies								
Positive	**112**	**11.0%**	6	4.3%	69	16.7%	37	7.9%
Negative	**647**	**63.6%**	87	63.0%	227	55.0%	333	71.5%
Unknown	**258**	**25.4%**	45	32.6%	117	28.3%	96	20.6%
Hepatitis B S-antigen								
Positive	**32**	**3.1%**	2	1.4%	14	3.4%	16	3.4%
Negative	**728**	**71.6%**	91	65.9%	282	68.3%	355	76.2%
Unknown	**257**	**25.3%**	45	32.6%	117	28.3%	95	20.4%
CD4+ cell count at diagnosis available^d^	**498**	**49.0%**	5	3.6%	203	49.2%	290	62.2%
≥500	**164**	**32.9%**	1		85	41.9%	78	26.9%
350–499	**90**	**18.1%**	3		39	19.2%	48	16.6%
200–349	**110**	**22.1%**	1		42	20.7%	67	23.1%
<200	**134**	**26.9%**			37	18.2%	97	33.4%
AIDS^e^	**154**	**15.1%**	30	21.7%	61	14.8%	63	13.5%
AIDS within 90 days of HIV	**72**	**7.1%**	3	2.2%	29	7.0%	40	8.6%
Deceased during follow-up	**63**	**6.2%**	18	13.0%	33	8.0%	12	2.6%

^a^If diagnosed before immigration, year of immigration. 1987**–**1997 includes 3 women diagnosed 1984**–**86

^b^Intravenous drug use

^c^Only two of the vertical transmissions and one of the blood transfusion transmissions occurred in Finland

^d^Measured in Finland 0**–**90 days after HIV diagnosis. Percentages reported of those with the CD4+ cell count at diagnosis available

^e^Diagnosed with an illness characteristic of AIDS (e.g., opportunistic infection). Does not include cases with CD4 + cell count < 200 in the absence of a concurrent AIDS-defining illness

Before HIV diagnosis, 160 women (15.7%) had 240 induced abortions, of which in 14 cases (5.8%) the HIV test was taken 0**–**10 days before the date of abortion (i.e., the HIV test was likely taken because of the planned abortion). After HIV diagnosis, 109 women (10.7%) had 156 induced abortions, of which in 20 cases (12.8%) the HIV diagnosis was made during the pregnancy based on the gestational age recorded at the time of the induced abortion.

**Fig. 2:**
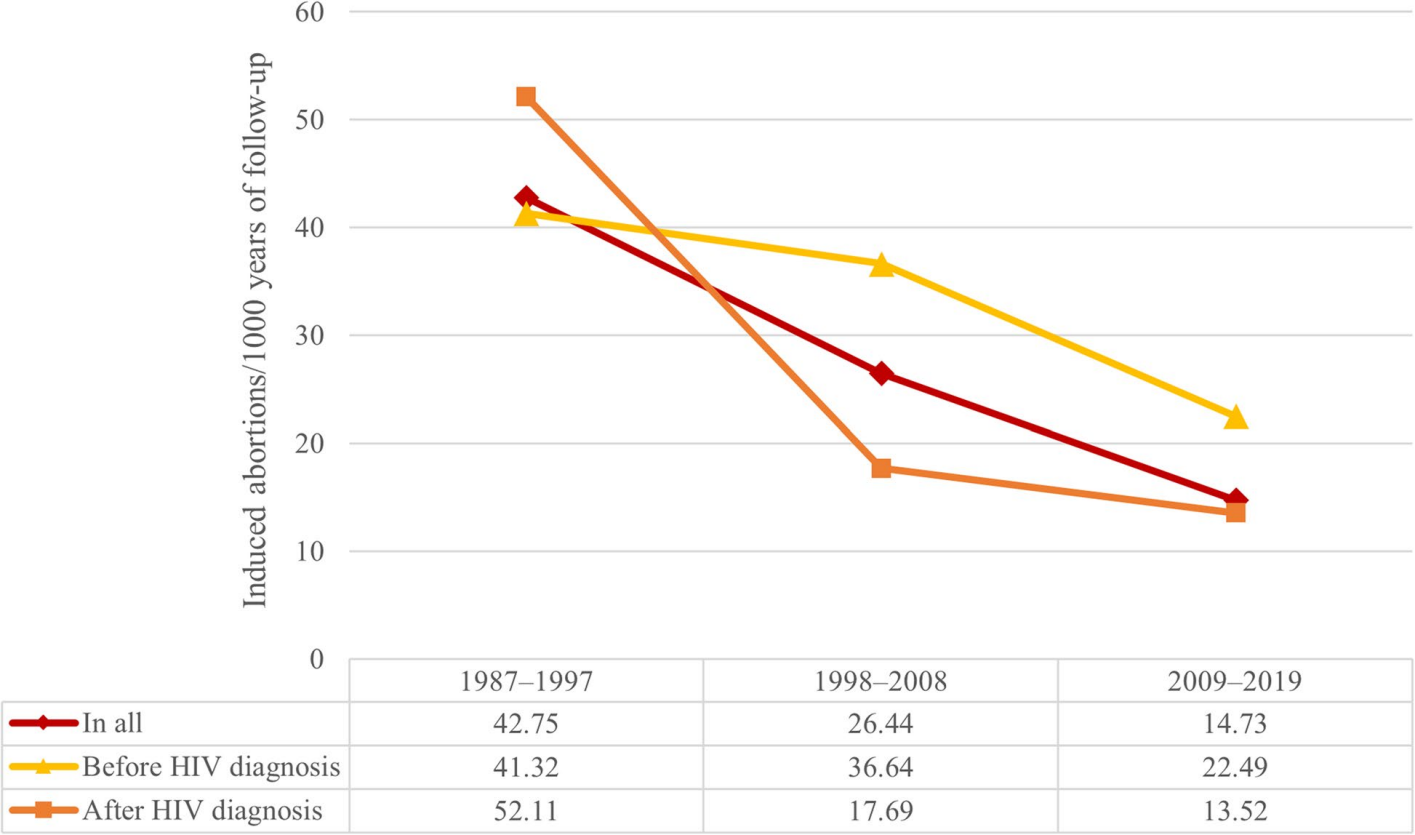
Induced abortion rate in the study population over time by year of follow-up (abortions/1000 follow-up years)

The induced abortion rate during 1987**–**2019 was 25.1 abortions/1000 follow-up years. It decreased from 42.8 abortions/1000 follow-up years during 1987**–**1997 to 14.7 abortions/1000 follow-up years during 2009**–**2019, with the decrease more prominent in induced abortions after diagnosis (Fig. 2).

**Fig. 3:**
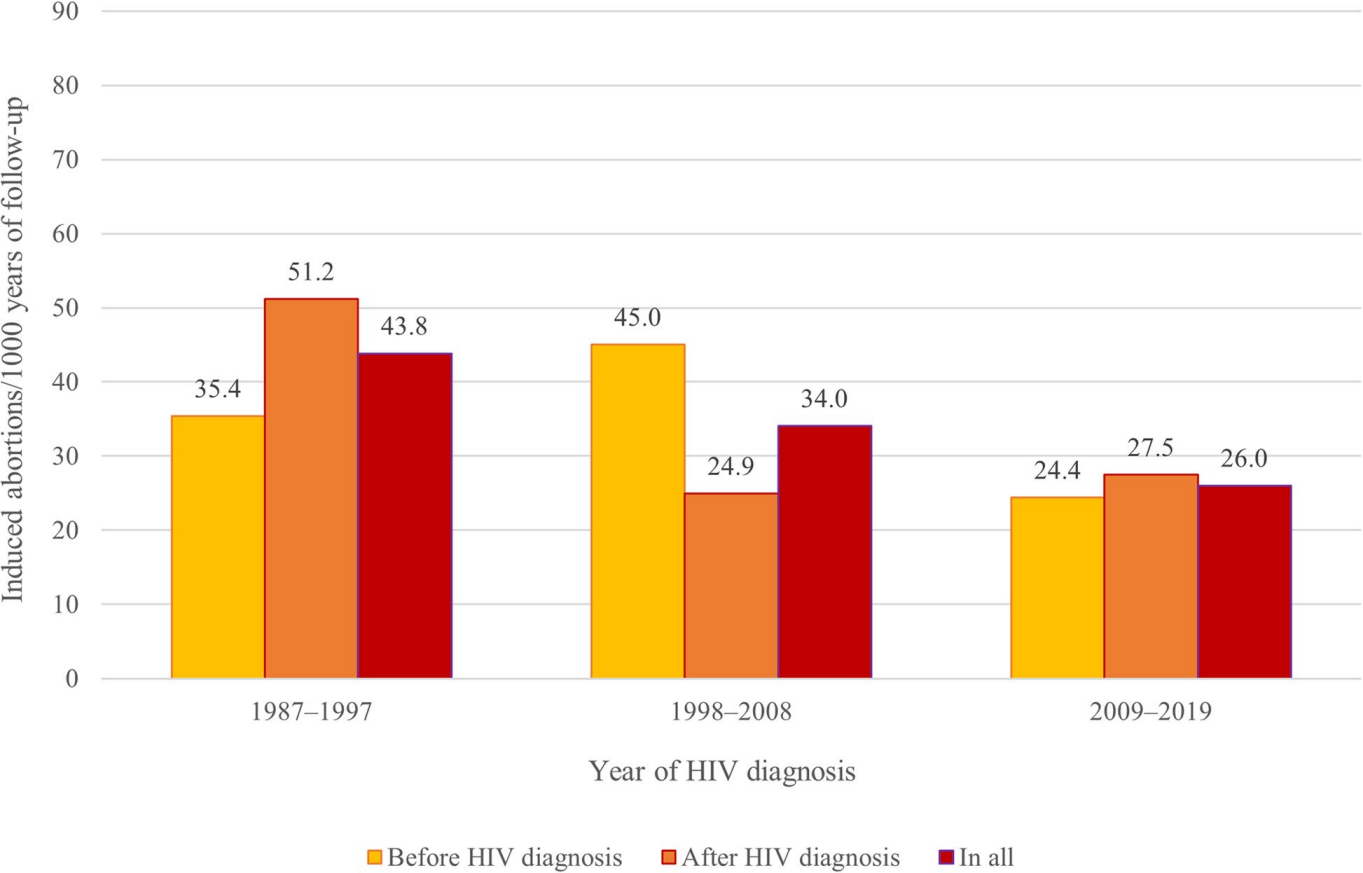
Induced abortions/1000 follow-up years 5 years before and after HIV diagnosis by year of diagnosis. Includes only the WLWH with follow-up time both before and after HIV diagnosis (*n* = 689)

To better assess the effect of the HIV diagnosis on the rate of induced abortion, we also examined separately the 5 years before and after HIV diagnosis for those who had follow-up time both before and after the diagnosis (i.e., diagnosed in Finland aged 15**–**49 years; *n* = 689). For those diagnosed in 1987**–**1997, the rate of abortions was higher after the HIV diagnosis (51.2 compared to 35.4 abortions/1000 follow-up years) while for those diagnosed in 1998**–**2008 the rate was lower after diagnosis (24.9 compared to 45.0 abortions/1000 follow-up years), and for those diagnosed in 2009**–**2019, there was little change before and after diagnosis (Fig. 3).

In the multivariable logistic regression model HIV diagnosis during or before pregnancy was associated with a decreased risk of induced abortion in 1998–2008 and 2009–2019 compared to 1987–1997 (Table 2). Increasing age was associated with a decreasing risk. In the model an increasing number of previous abortions or deliveries were both associated with an increased risk of induced abortion in women who were not born in Finland. Transmission route, hepatitis B and hepatitis C were not significant factors in any of the constructed models and are not included in the final models.

**Table 2:** Multivariable logistic regression model for induced abortion vs delivery 1987–2019

Variable	OR	95% Cl		*p*-value
		lower limit	upper limit	
Country of birth				
Other (reference Finland)	1.00	0.64	1.55	0.988
Age at conception (per year)	0.96	0.93	0.99	0.006
AIDS				
diagnosed during pregnancy	Too few cases			
diagnosed before pregnancy	2.61	0.97	7.03	0.057
Number of previous induced abortions				
Born in Finland	1.24	0.83	1.84	0.29
Not born in Finland	1.85	1.25	2.72	0.002
Number of previous deliveries				
Born in Finland	0.95	0.65	1.39	0.784
Not born in Finland	1.95	1.18	3.21	0.009
Year of conception				
1987–1997 (ref)	1			
1998–2008	1.39	0.89	2.17	0.153
2009–2019	0.67	0.32	1.42	0.300
HIV diagnosis during pregnancy^a^				
conception in:				
1987–1997	1.08	0.36	3.23	0.889
1998–2008	0.13	0.05	0.35	< 0.001
2009–2019	0.32	0.10	1.01	0.052
HIV diagnosed before pregnancy^a^				
conception in:				
1987–1997	1.92	0.85	4.34	0.117
1998–2008	0.30	0.17	0.55	< 0.001
2009–2019	0.34	0.15	0.76	0.008
Constant (clustering by woman)	2.03	0.94	4.38	0.07

Variables excluded from the model based on AIC, BIC and statistical non-significance *p* > 0.05: hepatitis B, hepatitis C, route of transmission. Interaction terms included in the model based on AIC, BIC and statistical significance *p* < 0.05: Relation of HIV diagnosis to period of conception, relations of country of birth to number of previous induced abortions and deliveries

^a^Diagnosis considered to be before pregnancy if diagnosis date (sampling date) more than 7 days prior to calculated conception date. If diagnosis date less than 7 days prior to conception date and either more than 10 days before abortion or at less than 23 (gestational) weeks, diagnosis was considered during pregnancy (could affect decision to terminate pregnancy). Consequently in case of giving birth, diagnosis ≥23 weeks was considered not known (= reference)

In analyzing factors associated with the risk of terminating a pregnancy specifically in pregnancies that began after HIV diagnosis in 1998–2019, the results were similar (Table 3). Not being born in Finland and previous induced abortions and pregnancies were associated with an increased risk of terminating a pregnancy, while increasing age was associated with a decreasing risk.

**Table 3:** Multivariable logistic regression for factors associated with terminating a pregnancy conceived after HIV diagnosis 1998–2019

Variable	OR	95% Cl		*p*-value
		lower limit	upper limit	
Country of birth				
Other (reference Finland)	3.09	1.55	6.19	0.001
Age at conception (per year)	0.95	0.90	0.997	0.037
AIDS				
diagnosed during pregnancy	Too few cases			
diagnosed before pregnancy	2.45	0.85	7.08	0.097
Number of previous induced abortions^b^	3.36	1.80	6.28	< 0.001
N of prev. i. abortions^2^ [squared] ^b^	0.83	0.69	1.00	0.051
Number of previous deliveries^b^	2.13	1.08	4.21	0.03
N of prev. deliveries^2^ [squared] ^b^	0.84	0.66	1.08	0.178
Constant (clustering by woman)	0.27	0.06	1.33	0.108

Variables excluded from the model based on AIC, BIC and statistical non-significance p > 0.05: hepatitis B, hepatitis C and route of transmission

^b^Calculated increase in OR until 3–4 previous induced abortions and 2–3 previous deliveries after which the OR decreases

There were 68 women who had 88 induced abortions during 1987–2014 within 5 years before HIV diagnosis. During the same years, 304,985 induced abortions were performed in Finland, which makes the maximum prevalence of undiagnosed HIV 0.29/1000 abortions for those years. By using the CD4+ cell count thresholds, we estimated that of the 40 abortions where the woman had a CD4+ cell count at diagnosis available, HIV was undiagnosed at 11 abortions (28%). Assuming this percentage would hold true for the 88 abortions, the minimum prevalence of undiagnosed HIV would be 0.08/1000 induced abortions for 1987–2014 (Table 4).

**Table 4:** Analysis for prevalence of undiagnosed HIV at induced abortions

	Assumed maximum time from transmission to symptoms that prompt HIV testing		
	5 years	10 years	15 years
Time considered^a^	**1987–2014**	1987–2009	1987–2004
**Maximum estimate:**			
Induced abortions in Finland^b^	**304,985**	253,739	200,518
WLWH with i.abortion before HIV diagnosis	**68**	100	110
Number of i.abortions before HIV diagnosis	**88**	136	152
Undiagnosed HIV/1000 induced abortions	**0.29**	0.54	0.76
**Minimum estimate based on CD4+ cell count:**			
WLWH with CD4+ count at diagnosis available^c^	**32**	50	56
Abortions with CD4+ count at diagnosis available	**40**	65	71
WLWH estimated undiagnosed at ≥1 abortion^d^	**10**	19	20
Abortions as undiagnosed based on CD4 count	**11**	21	22
Undiagnosed HIV/abortions with CD4+ count avail.	**28%**	32%	31%
Undiagnosed HIV/1000 induced abortions	**0.08**	0.17	0.23
**Estimate for foreign-born women:**			
Induced abortions among foreign-born in Finland^e^	**24,395**	17,761	12,030
Foreign-born WLWH with abortion	**25**	28	23
Number of abortions among foreign-born WLWH	**29**	35	30
Undiagnosed HIV/1000 induced abortions	**1.19**	1.97	2.49
**Estimate for women with previous i. abortion:**			
Abortions in Finland with previous i. abortions^b^	**97,956**	79,118	60,229
WLWH with prev. Abortion at the time of abortion	**31**	41	43
Abortions of WLWH with prev. abortions	**40**	54	50
Undiagnosed HIV/1000 induced abortions	**0.41**	0.68	0.83
**Estimate for women with previous deliveries:**			
Abortions in Finland with previous deliveries^b^	**150,647**	125,691	100,403
WLWH with abortion and prev. delivery in the data	**22**	37	42
Abortions of WLWH with prev. delivery in the data	**35**	54	57
Undiagnosed HIV/1000 induced abortions	**0.23**	0.43	0.57

^a^To ensure all who had an induced abortion would have been diagnosed before study end-point

^b^Data from THL open data [19]

^c^Measured in Finland 0–90 days after HIV diagnosis

^d^Used thresholds based on Lodi et al., [18] CD4+ cell count at diagnosis → estimated time since transmission

for 5 year-limit: ≥500 → 6 months, 350–499 → 1 year, 200–349 → 3 years, < 200 → 5 years

for 10-year-limit: ≥500 → 1 year, 350–499 → 3 years, 200–349 → 8 years, < 200 → 10 years

for 15-year-limit: ≥ 500 → 2 years, 350–499 → 6 years, 200–349 → 12 years, < 200 → 15 years

^e^Calculated by roughly estimated proportions from [16, 20]: 8% for 5 year limit, 7% for 10 year limit, 6% for 15 year limit

The estimated maximum prevalence for women not born in Finland, women with previous induced abortions, and women with previous deliveries were 1.19, 0.41 and 0.23/1000 induced abortions, respectively. Of those women not born in Finland who had an induced abortion 0–5 years before HIV diagnosis, all but one were born in countries where the prevalence of HIV is considered high enough (> 1%) to warrant opt-out screening of HIV for refugees, asylum seekers and immigrants after arrival in Finland [21, 22].

Because both the time frame for development of symptomatic HIV infection prompting testing and the rate of decline for CD4+ cells are varied and uncertain, we did a sensitivity analysis by changing the time frame and thresholds to 10 and 15 years before HIV diagnosis and the results did not change significantly.

## Discussion

### Main findings

The rate of induced abortions among WLWH in Finland has decreased from 42.8 to 14.7/1000 follow-up years from 1987 to 1997 to 2009–2019, with the change more pronounced in abortions after HIV diagnosis. In our predictive regression model, after 1997 being diagnosed with HIV was not associated with an increased risk of terminating a pregnancy. Factors associated with terminating a pregnancy among WLWH in our model were younger age, country of birth other than Finland, and having previous induced abortions or deliveries. Estimated prevalence of undiagnosed HIV at induced abortions in Finland 1987–2014 was 0.008–0.029%.

### Strengths and limitations

Strengths of the study are nationwide coverage and the use of register data over a long, 33-year follow-up period. The study population comprises all WLWH in Finland since the beginning of the HIV epidemic, and linkage between registers is accurate as the PIC is used as an identification in all health care contacts in Finland. The only WLWH missing from the study population are those with no valid PIC, who are mostly short-time visitors unlikely to contribute many events or much follow-up time in the data. The Register on Induced Abortions and Sterilizations was recently estimated to include 97% of the induced abortions [23]. Since it is easy to access induced abortion in public health care, there should be no underestimation due to clandestine abortions.

The major weakness is that only data from Finland were available. Abortions or deliveries before immigration are not included and for immigrants, the data are included only after receipt of a valid PIC (generally upon a permit to stay exceeding 1 year). The emigration (and to some extent, immigration) dates are incomplete especially for the earlier years of the follow-up. Because our data had no socioeconomic factors we considered relevant for determining reasons for having an induced abortion, the regression models were constructed for predictive aims only and causality of the factors included cannot be inferred from the models. Additionally, the study population consists of only 1017 women and so statistical modelling for some factors with several relevant variables is not accurate enough to examine small associations. Order of previous pregnancy outcomes or time since last pregnancy were not considered in model construction. Association of HIV diagnosis with risk of terminating an on-going pregnancy might be biased by the fact that there has been a national opt-out antenatal screening of all pregnant women planning to deliver since 1998, but HIV testing at induced abortions is not routine.

### Interpretation

To our knowledge, this is the first nationwide study on induced abortions among WLWH covering over 30 years. The rates of induced abortions among WLWH both before and after HIV diagnosis are approaching the rate in the Finnish general population (7.7/1000 women of 15–49 years of age in 2019, steadily decreasing from 10.3 in 1987) [24]. The slightly higher rate among WLWH might be explained, besides differences in calculating time at risk, by the higher proportion of WLWH in follow-up at the age of 20–30 years and the higher proportion of immigrants, as they are at higher risk than Finnish-born individuals [16]. The decrease in induced abortion rate was similar to the decrease seen in an Italian cohort from 1980s to 2010 [15] but differed from an US cohort where no change was seen in 1994–1997 to 2006–2012 [10].

The decrease in the rate of induced abortions was more pronounced after HIV diagnosis and after 1997 being diagnosed with HIV was associated with a decreased risk of terminating a pregnancy in our predictive model. This might reflect the trust in diminished risk of vertical transmission of the women and their caregivers, as well as the possibilities of WLWH to discuss their reproductive plans and contraception with their caregivers.

Of the WLWH in Finland, 11% have had an induced abortion after being diagnosed, a proportion similar to some previous studies [15, 25, 26]. Similar to our study, younger age and previous pregnancies have been associated with an increased risk of induced abortion in two previous cohort studies among WLWH in Italy and the US [10, 15]. In contrast to these studies where migration status or ethnicity were not significantly associated with the risk of induced abortion, in our model not being born in Finland was associated with an increased risk. This is however consistent with the Finnish general population, where first-generation immigrants had a higher risk of induced abortion than Finnish-born women in a recent national register study [16].

The estimated prevalence of undiagnosed HIV at induced abortion was 0.08–0.29/1000 abortions (0.008–0.029%) during 1987–2014. A prevalence of 0.1–1.0% is generally considered cost effective for testing, though some have suggested a prevalence as low as 0.05% [27–29]. Therefore, although the prevalence of the most recent years cannot be determined for certain without implementing routine testing, it is reasonable to assume that routine testing of all women having an induced abortion in Finland is not cost-effective if the limit is set at 0.1%. This is in contrast with estimates for some other high-income countries, where the HIV prevalence is higher and routine testing at induced abortions may be recommendable based on the same criteria [30]. However, in Finland the prevalence of undiagnosed HIV is likely higher among women not born in Finland (0.12%) and more specifically, women born in countries with a high HIV prevalence; an induced abortion would therefore be an event that could prompt offering HIV testing to those with pre-existing risk factors, including country of birth. The prevalence of undiagnosed HIV for those with previous induced abortions or deliveries is not high enough to warrant routine testing when using the 0.1% limit.

## Conclusions

The rate of induced abortions among WLWH in Finland has decreased markedly in three decades, approaching the rate among the general population. In our model, being diagnosed with HIV was not associated with an increased risk of terminating a pregnancy after effective cART to prevent vertical transmission became available. However, since every woman living with HIV in Finland meets her doctor once or twice a year with the possibility to discuss family plans and contraception, one might assume the rate of induced abortions among WLWH to be even lower. Caregivers in Finland should remember to discuss the wishes of WLWH regarding family planning at every follow-up appointment, and especially with younger women, women with previous pregnancies, and women who were not born in Finland.

Routine testing of all women at induced abortion is not likely to be cost-effective in Finland due to low HIV prevalence, but testing could be offered to women with pre-existing risk factors, such as being born in a country with a high HIV prevalence.

## Data Availability

The data that support the findings of this study are available from Data Permit Authority Findata that works in conjunction with the Finnish Institute for Health and Welfare but restrictions apply to the availability of these data, which were used under license for the current study, and so are not publicly available. For more information on how the data may be requested please contact the corresponding author (mikaela.mutru@helsinki.fi).

## Abbreviations

WLWH: Women living with HIV
ART: Antiretroviral treatment
cART: Combined antiretroviral treatment
IDU: Intravenous drug use
PIC: Personal identification code
FINHIV: Finnish HIV Quality of Care Register
THL: Finnish Institute for Health and Welfare
